# Eicosapentaenoic acid (EPA)-induced inhibitory effects on porcine coronary and cerebral arteries involve inhibition of prostanoid TP receptors

**DOI:** 10.1038/s41598-022-16917-6

**Published:** 2022-07-27

**Authors:** Kento Yoshioka, Keisuke Obara, Shunya Oikawa, Kohei Uemura, Akina Yamaguchi, Kazuki Fujisawa, Hitomi Hanazawa, Miki Fujiwara, Taison Endoh, Taichi Suzuki, Montserrat De Dios Regadera, Daichi Ito, Noboru Saitoh, Yutaka Nakagome, Toma Yamashita, Mayu Kiguchi, Yuka Saito, Yuri Nakao, Hinako Miyaji, Guanghan Ou, Keyue Xu, Yoshio Tanaka

**Affiliations:** Department of Chemical Pharmacology, Faculty of Pharmaceutical Sciences, Toho University, Miyama 2-2-1, Funabashi-City, Chiba 274-8510 Japan

**Keywords:** Receptor pharmacology, Cardiovascular biology

## Abstract

This study was performed to elucidate whether eicosapentaenoic acid (EPA) suppresses spasm-prone blood vessel contractions induced by a thromboxane mimetic (U46619) and prostaglandin F_2α_ (PGF_2α_) and determine whether the primary target of EPA is the prostanoid TP receptor. Accordingly, we assessed: (1) the tension changes in porcine basilar and coronary arteries, and (2) changes in the Fura-2 (an intracellular Ca^2+^ indicator) fluorescence intensity ratio at 510 nm elicited by 340/380 nm excitation (F340/380) in 293T cells expressing the human TP receptor (TP-293T cells) and those expressing the human prostanoid FP receptor (FP-293T cells). EPA inhibited both porcine basilar and coronary artery contractions induced by U46619 and PGF_2α_ in a concentration-dependent manner, but it did not affect the contractions induced by 80 mM KCl. EPA also inhibited the increase in F340/380 induced by U46619 and PGF_2**α**_ in TP-293T cells. In contrast, EPA showed only a marginal effect on the increase in F340/380 induced by PGF_2α_ in FP-293T cells. These findings indicate that EPA strongly suppresses the porcine basilar and coronary artery contractions mediated by TP receptor and that inhibition of TP receptors partly underlies the EPA-induced inhibitory effects on these arterial contractions.

## Introduction

Eicosapentaenoic acid (EPA) and docosahexaenoic acid (DHA) are representative n-3 polyunsaturated fatty acids (PUFAs). Many epidemiological studies and intervention studies have shown that EPA prevents and improves cardiovascular conditions, for example, by preventing coronary artery diseases and strokes, ameliorating hypertension, and exhibiting antiplatelet effects^1^. The clinical applications of EPA-related medicines also indicate the importance of the preventive/therapeutic effects of n-3 PUFAs against cardiovascular diseases. Several recent clinical studies (JELIS, REDUCE-IT, and EVAPORATE) have shown that EPA ethyl ester reduced the risk of cardiovascular disease. In contrast, some clinical studies using the combination of EPA/DHA (OMEMI and STRENGTH) or EPA/DHA ethyl ester (VITAL) have shown no significant effects^2^. Although this difference may be due to differences in the clinical study protocols, EPA may be more effective than a combination of EPA/DHA against cardiovascular disease. In Japan, Epadel®, an ethyl ester preparation of EPA, and Lotriga®, a combination of EPA ethyl ester and DHA ethyl ester, are clinically used. Epadel® is used to treat arteriosclerosis obliterans-associated ulcers, pain and coldness, and hyperlipidemia^3^, and Lotriga® is used to treat hyperlipidemia^3^. n-3 PUFAs, including EPA, serve as substrates for arachidonic acid-metabolizing enzymes and can be competitively metabolized with arachidonic acid; thus, suppression of the production of constrictor prostaglandins and/or production of relaxant prostaglandins are speculated to be the mechanisms by which EPA prevents and improves cardiovascular diseases^4^. In fact, regarding the possible mechanisms of the antiplatelet action of Epadel®, EPA is reported to be converted to thromboxane A_3_ (TXA_3_), which lacks platelet aggregation activity, and prostaglandin I_3_ (PGI_3_), whose antiplatelet action is almost comparable to that of prostaglandin I_2_ (PGI_2_)^5,6^. However, while many studies of the effects of EPA on the cardiovascular system have examined the long-term intake effects of EPA on humans or animals, studies on the immediate effects on isolated blood vessels are very limited.

Basic and clinical research has indicated that thromboxane A_2_ (TXA_2_) is a substantial inducer of cerebral vasospasm and angina. For example, (1) after subarachnoid hemorrhage, release of the TXA_2_ metabolite thromboxane B_2_ (TXB_2_) and thromboxane formation capacity in cerebral arteries were significantly higher in patients with severe vasospasm than in those without vasospasm^7^; (2) In patients with spontaneous angina, TXB_2_ concentrations were significantly higher in the coronary circulation than in the aortic circulation during the angina episode^8^; (3) An increased number of coronary arterial TP receptors have been detected after acute myocardial infarction^9^; and (4) TP receptor knockout decreased the incidence of arteriosclerotic lesions in mice^10^. These findings indicate that TXA_2_ plays a key role in the occurrence of cerebral and coronary arterial spasms.

In this regard, we found that EPA and DHA immediately and selectively suppress the contractions induced by a thromboxane mimetic (U46619) and prostaglandin F_2α_ (PGF_2α_) in rat aorta and mesenteric artery. These findings indicate that suppression of TXA_2_- and PGF_2α_-induced vascular contractions is partly responsible for the cardioprotective effects of EPA and DHA^11,12^. We also recently showed that DHA potently inhibits porcine basilar and coronary artery contractions induced by U46619/PGF_2α_, and that prostanoid TP receptors might be targets for DHA^13^. In the present study, we show that EPA, like DHA, suppresses the contraction of coronary and cerebral arteries by the action of U46619/PGF_2α_ by inhibiting the TP receptor.

## Results

### Effects of EPA on U46619/PGF_2α_/KCl-induced porcine basilar artery tonic contractions

**Figure 1:**
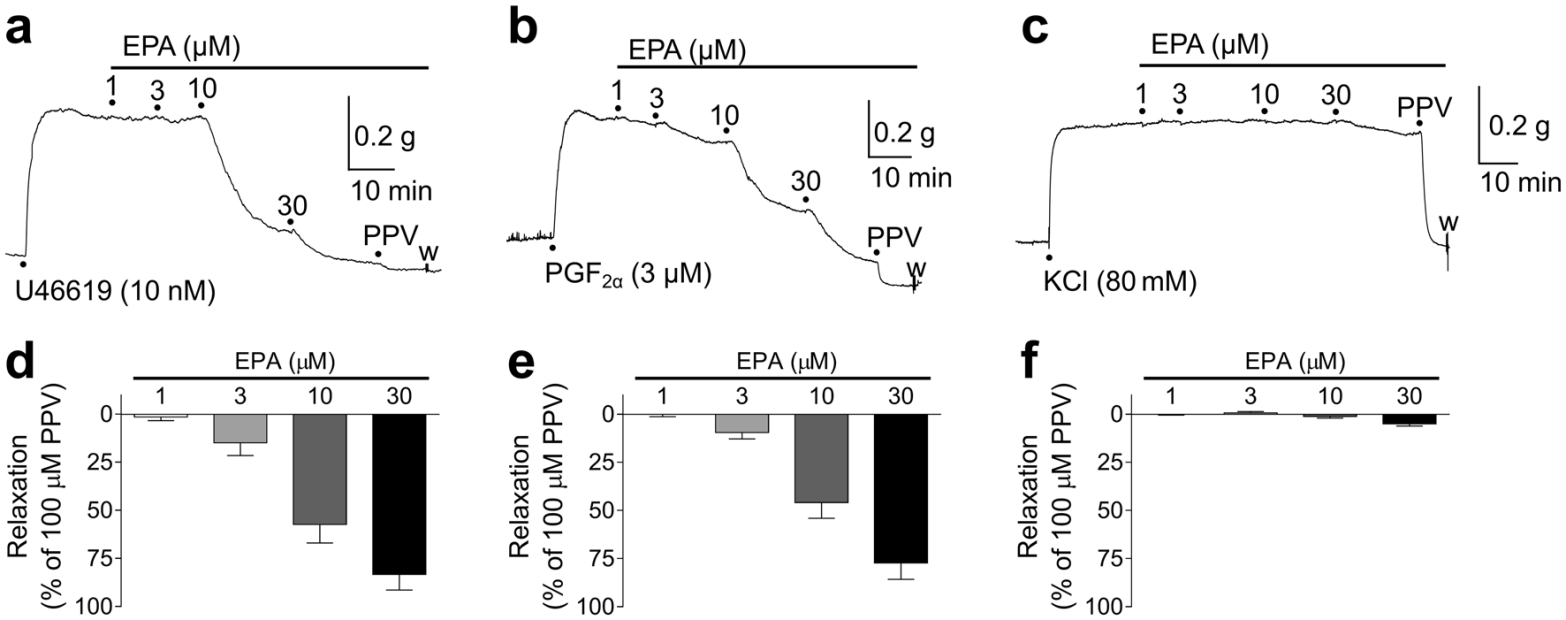
Representative traces showing relaxant responses to 1–30 μM eicosapentaenoic acid (EPA) on porcine basilar artery tonic contractions induced by 10 nM U46619 (**a**), 3 μM prostaglandin (PG) F_2α_ (**b**), and 80 mM KCl (**c**); quantified data of relaxant responses to 1–30 μM EPA on the contractions induced by 10–30 nM U46619 (**d**), 3–6 μM PGF_2α_ (**e**), and 80 mM KCl (**f**). Data are presented as mean ± SEM (n = 7 (**d**,**e**) and n = 6 (**f**)). ●: each drug application. PPV: 100 μM papaverine.

Representative traces of the effects of EPA on porcine basilar artery contractions induced by 10–30 nM U46619, 3–6 μM PGF_2α_, and 80 mM KCl are shown in Fig. 1a–c, respectively, whereas the corresponding quantified data are shown in Fig. 1d–f. EPA (1–30 μM) suppressed the contractions induced by U46619 (Fig. 1a,d) and PGF_2α_ (Fig. 1b,e) in a concentration-dependent manner. EPA was prone to suppress contractions induced by U46619 more strongly than those induced by PGF_2α_; specifically, 30 μM EPA inhibited 83.2 ± 8.2% [mean ± standard error of the mean (SEM), n = 7)] and 77.2 ± 8.5% (mean ± SEM, n = 7) of the contractions induced by U46619 and PGF_2α_, respectively. In contrast, EPA barely inhibited the contractions generated by 80 mM KCl (Fig. 1c,f).

#### Effects of EPA on U46619/PGF2α/KCl-induced porcine coronary artery tonic contractions

**Figure 2:**
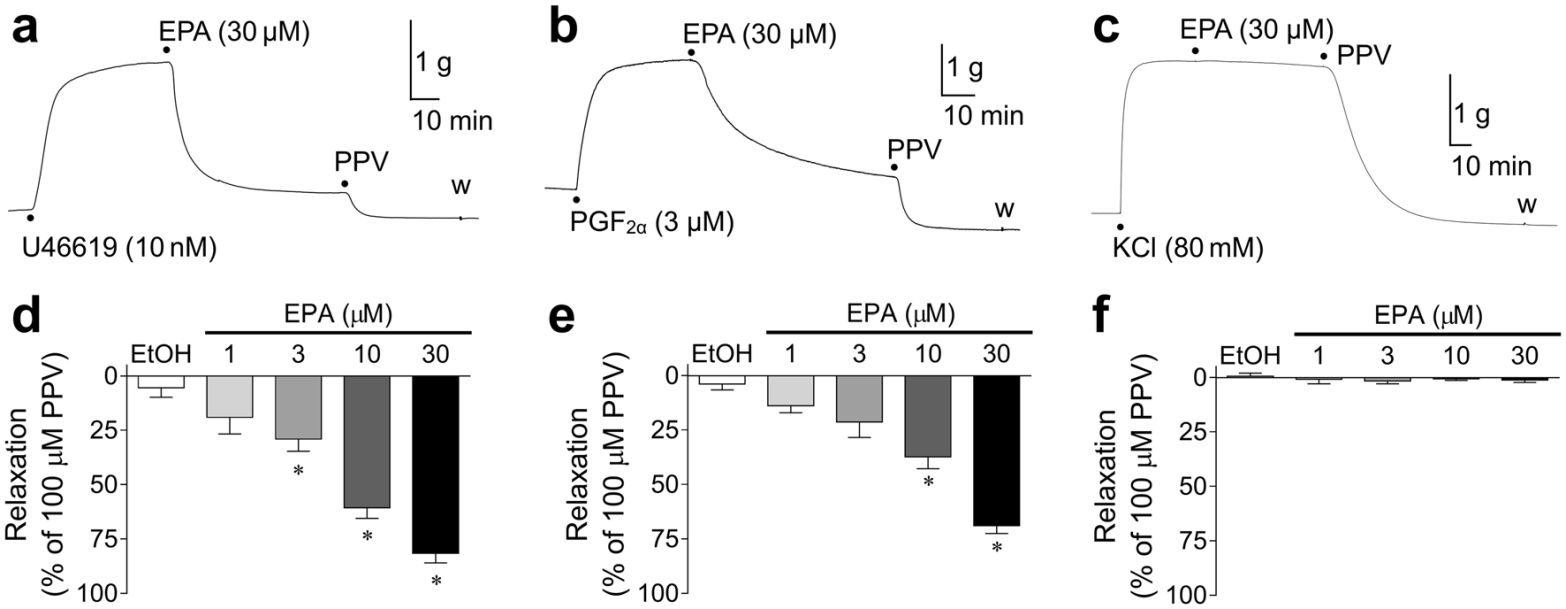
Representative traces showing relaxant responses to 30 μM eicosapentaenoic acid (EPA) on porcine coronary artery tonic contractions induced by 10 nM U46619 (**a**), 3 μM prostaglandin (PG) F_2α_ (**b**), and 80 mM KCl (**c**); quantified data of relaxant responses to 1–30 μM EPA and 0.1% ethanol (EtOH) on the contractions induced by 10–30 nM U46619 (**d**), 3–30 μM PGF_2α_, (**e**), and 80 mM KCl (**f**). Data are presented as mean ± SEM [n = 5–12 (**d**), n = 6–13 (**e**), and n = 6–10 (**f**)]. ●: each drug application. PPV: 100 μM papaverine. *P < 0.05 versus EtOH (Dunnett's test after one-way ANOVA).

Representative traces of the effects of 30 μM EPA on porcine coronary artery tonic contractions induced by 10 nM U46619, 3 μM PGF_2α_, and 80 mM KCl are shown in Fig. 2a–c, respectively. Figure 2d–f shows the quantified data of the concentration-dependent relaxant effects of 1–30 μM EPA on the contractions induced by 10–30 nM U46619 (Fig. 1d), 3–10 μM PGF_2α_ (Fig. 2e), and 80 mM KCl (Fig. 2f). EPA (1–30 μM) suppressed the contractions induced by U46619 (Fig. 2a,d) and PGF_2α_ (Fig. 2b,e) in a concentration-dependent manner. EPA inhibited the contractions induced by U46619 more strongly than those induced by PGF_2α_; specifically, 30 μm EPA inhibited 81.6 ± 4.3% (mean ± SEM, n = 12) and 68.9 ± 3.6% (mean ± SEM, n = 13) of the U46619- and PGF_2α_-induced contractions, respectively. In contrast, EPA barely inhibited the contractions induced by 80 mM KCl (Fig. 2c,f).

#### Effects of EPA on acetylcholine (ACh)/histamine (His)/serotonin (5-HT)-induced porcine coronary artery phasic contractions

**Figure 3:**
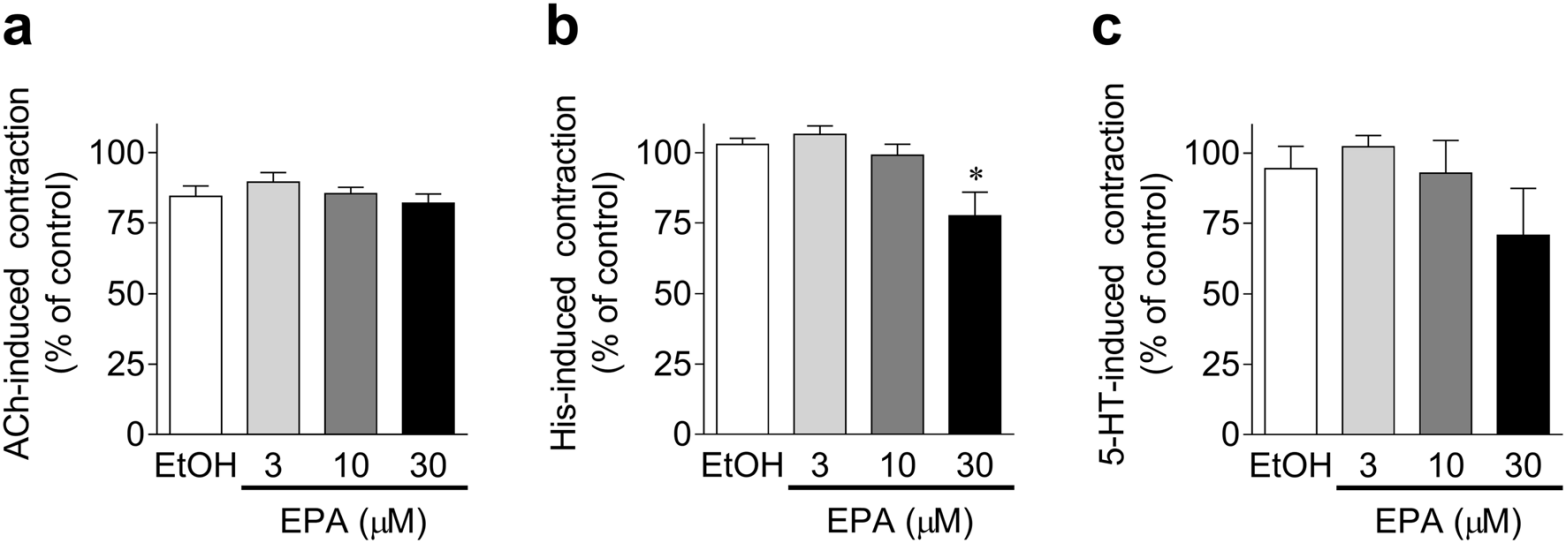
Quantified data of effects of 3–30 μM eicosapentaenoic acid (EPA) and 0.1% ethanol (EtOH) on porcine coronary artery phasic contractions induced by 3 μM acetylcholine (ACh, **a**), 10 μM histamine (His, **b**), and 30 μM serotonin (5-HT, **c**). Data are presented as mean ± SEM [n = 6 (**a**) and n = 5 (**b**,**c**)]. *P < 0.05 versus EtOH (Dunnett's test after one-way ANOVA).

The concentration-dependent effects of 3–30 μM EPA on porcine coronary artery phasic contractions induced by 3 μM ACh, 10 μM His, and 30 μM 5-HT are shown in Fig. 3a–c, respectively. EPA (3–30 μM) did not significantly suppress the contractions induced by ACh (Fig. 3a). In contrast, 30 μM EPA weakly (~ 20–30%) suppressed His (Fig. 3b)- and 5-HT (Fig. 3c)-induced contractions, although only the former showed statistical significance.

#### Effects of SQ 29,548 and SQ 29,548 plus EPA on U46619- and PGF_2α_-induced tonic contractions in porcine basilar and coronary arteries

**Figure 4:**
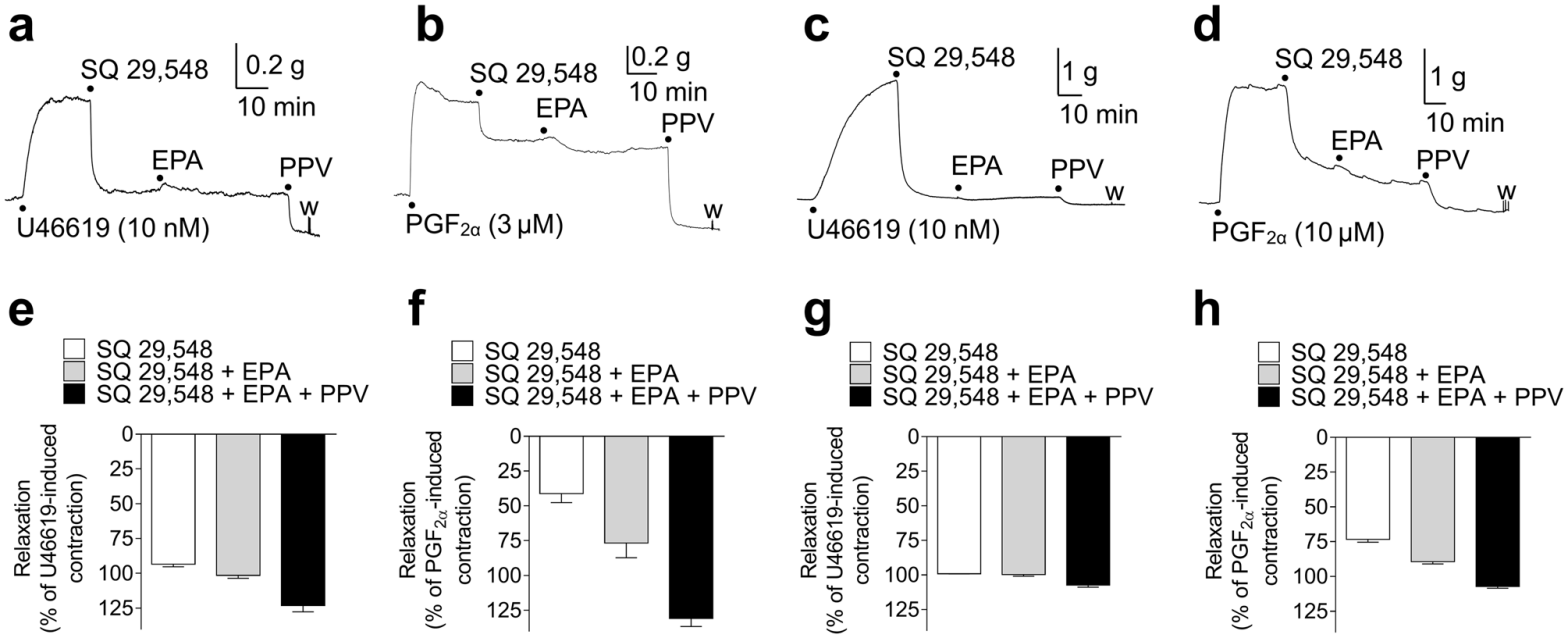
Representative traces showing relaxant responses to 1 μM SQ 29,548 and 30 μM eicosapentaenoic acid (EPA) on porcine basilar (**a**,**b**) and coronary (**c**,**d**) artery tonic contractions induced by 10 nM U46619 (**a**,**c**) and 3 μM/10 μM prostaglandin (PG) F_2α_ (**b**/**d**); quantified data of relaxant responses to 1 μM SQ 29,548 and 30 μM EPA on porcine basilar (**e**,**f**) and coronary (**g**,**h**) artery tonic contractions induced by 10–30 nM U46619 (**e**,**g**) and 3–6 μM/10–30 μM PGF_2α_ (**f**/**h**). Data are presented as mean ± SEM [n = 5 (**e**,**g**), n = 7 (**f**), and n = 11 (**h**)]. ●: each drug application. PPV: papaverine (100 μM).

Figure 4 shows representative traces (Fig. 4a–d) and quantified data (Fig. 4e–h) of the effects of 1 μM SQ 29,548 (a selective TP receptor antagonist) and 30 μM EPA on porcine basilar (Fig. 4a,b,e,f) and coronary artery (Fig. 4c,d,g,h) contractions induced by 10–30 nM U46619 (Fig. 4a,c,e,g) and 3–30 μM PGF_2α_ (Fig. 4b,d,f,h). In both porcine basilar and coronary arteries, the contractions induced by U46619 were almost completely inhibited by 1 μM SQ 29,548, and the remaining contractions were slightly inhibited by 30 μM EPA (Fig. 4a,c,e,g). In porcine basilar arteries, PGF_2α_-induced contractions were partly inhibited (~ 40%) by 1 μM SQ 29,548, and the remaining contractions were further inhibited (~ 75%) by 30 μM EPA (Fig. 4b,f). In porcine coronary arteries, the contractions induced by PGF_2α_ were strongly inhibited (~ 70%) by 1 μM SQ 29,548, and the remaining contractions were further inhibited (~ 90%) by 30 μM EPA (Fig. 4d,h).

#### Effects of EPA on increases in Fura-2 (an intracellular Ca^2+^ indicator) fluorescence intensity ratio (F340/380) induced by U46619 and PGF_2α_ in 293T cells expressing the human TP receptor (TP-293T cells)

**Figure 5:**
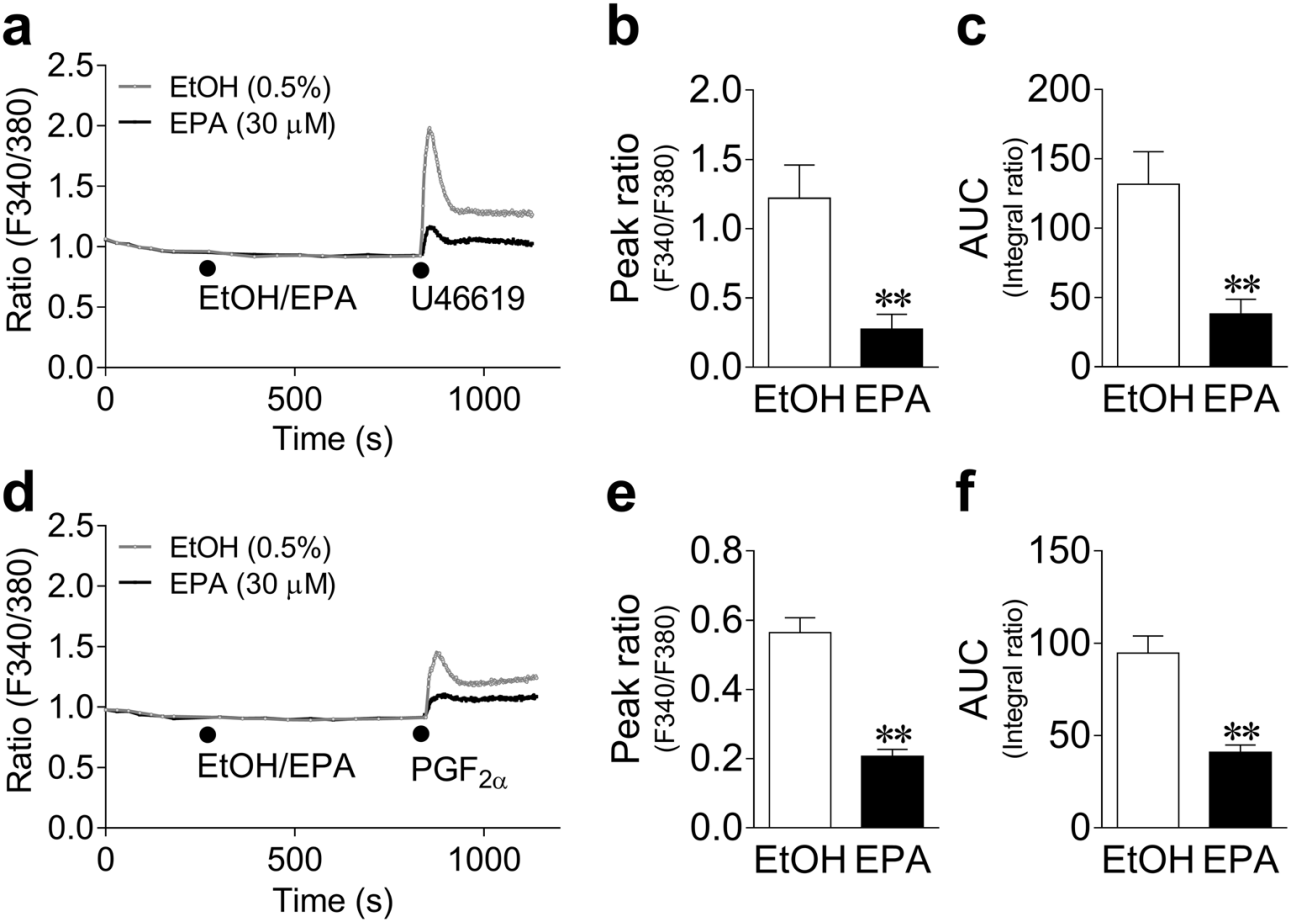
Changes in Fura-2 (an intracellular Ca^2+^ indicator) fluorescence intensity ratio at 510 nm elicited by 340/380 nm excitation (F340/380) induced by 10 nM U46619 (**a**) and 3 μM prostaglandin (PG) F_2α_ (**d**) in the presence of 30 μM eicosapentaenoic acid (EPA) or 0.5% ethanol (EtOH) in cells expressing the human prostanoid TP receptor; quantified data of the F340/380 peak after application of U46619 (**b**) or PGF_2α_ (**e**) and the F340/380 integral (area under the curve, AUC) within 5 min of application of U46619 (**c**) or PGF_2α_ (**f**). Changes in F340/380 are presented as means only, and quantified data are presented as mean ± SEM (each n = 10). ●: each drug application. **P < 0.01 versus EtOH (Student's *t*-test).

The effects of EPA on the increases in F340/380 induced by U46619 and PGF_2α_ in TP-293T cells are shown in Fig. 5a–c and Fig. 5d–f, respectively. EPA (30 μM) largely suppressed the increased levels of F340/380 induced by both 10 nM U46619 (Fig. 5a**)** and 3 μM PGF_2α_ (Fig. 5d). The inhibitory effects of EPA on the increases in Ca^2+^ concentrations induced by U46619 and PGF_2α_ are summarized in the quantified data in Fig. 5, which show that 30 μM EPA significantly suppressed both the peak F340/380 after U46619 (Fig. 5b)/PGF_2α_ (Fig. 5e) application, and F340/380 integral for 5 min (the area under the curve, AUC) after U46619 (Fig. 5c)/PGF_2α_ (Fig. 5f) application. The TP receptor antagonist SQ 29,548 (1 μM) almost completely suppressed the increases in F340/380 induced by U46619 (10 nM) (Supplementary Fig. S1a, b).

#### Effects of EPA on increases in F340/380 induced by PGF_2α_ in 293T cells expressing the human prostanoid FP receptor (FP-293T cells)

**Figure 6:**
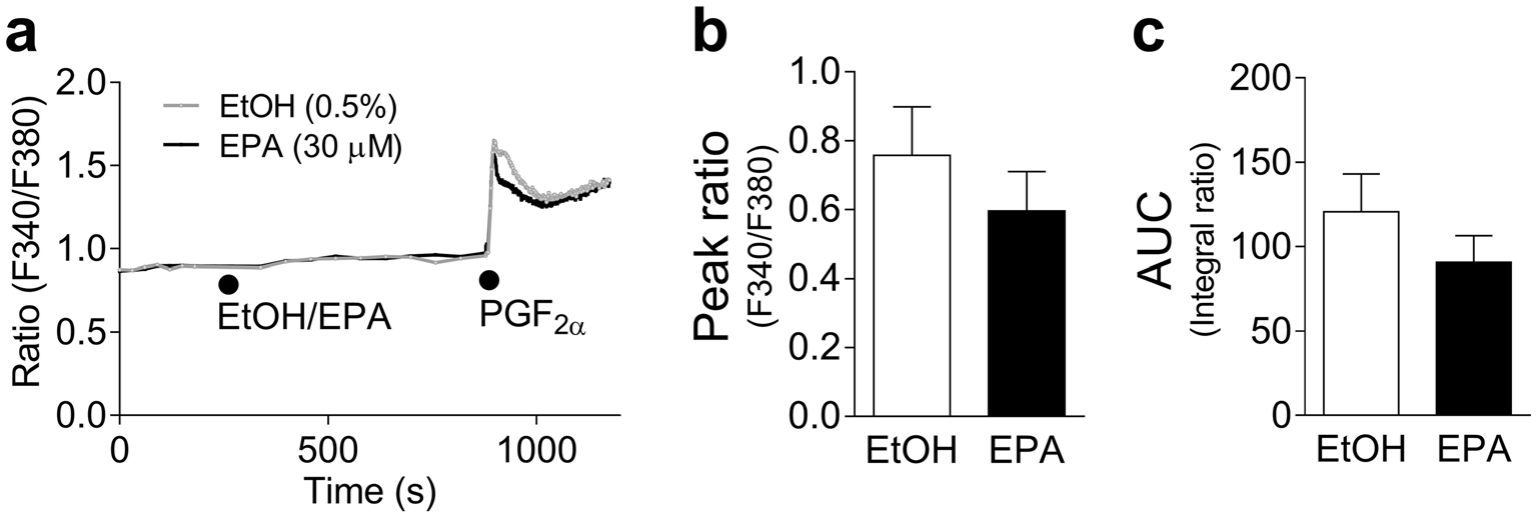
Changes in Fura-2 (an intracellular Ca^2+^ indicator) fluorescence intensity ratio at 510 nm elicited by 340/380 nm excitation (F340/380) induced by 3 μM prostaglandin (PG) F_2α_ in the presence of 30 μM eicosapentaenoic acid (EPA) or 0.5% ethanol (EtOH) (**a**); quantified data of the F340/380 peak (**b**) and the F340/380 integral (area under the curve, AUC) within 5 min of PGF_2__α_ application (**c**) in cells expressing the human prostanoid FP receptor. Changes in F340/380 are presented as means only, and quantified data are shown as mean ± SEM (each n = 10). ●: each drug application.

The effects of EPA on the increases in F340/380 induced by PGF_2α_ in FP-293T cells (Fig. 6a), and the quantified data of the peak F340/380 after PGF_2α_ application (Fig. 6b) and of the AUC (Fig. 6c) are shown in Fig. 6. EPA (30 μM) showed only a marginal effect on both the peak F340/380 (Fig. 6b) and AUC (Fig. 6c). The FP receptor antagonist phloretin (100 μM) significantly suppressed the increase in F340/380 induced by PGF_2α._ (3 μM) (Supplementary Fig. S1c,d).

#### Effects of EPA on concentration–response curves (CRCs) of porcine basilar and coronary artery contractions induced by U46619

**Figure 7:**
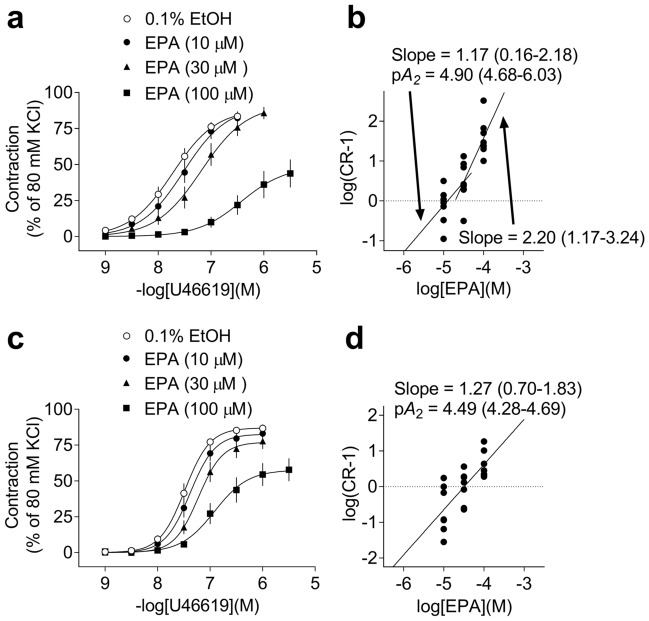
Quantified data of inhibitory effects of 10–100 μM eicosapentaenoic acid (EPA) on the concentration–response curves (CRCs) for porcine basilar (**a**) and coronary (**c**) artery contractions induced by U46619 and Schild plot analysis of EPA versus U46619 in porcine basilar (**b**) and coronary (**d**) arteries. CRCs are shown as mean ± SEM [n = 10 (**a**) and n = 8 (**c**)] and Schild plots are shown as individual data points [n = 24 (**b**) and n = 21 (**d**)]. The slope and p*A*_2_ values are shown as means with 95% confidence intervals.

The effects of 10–100 μM EPA on the CRCs of contractions induced by U46619 in porcine basilar and coronary arteries are shown in Fig. 7a–d, respectively. In both basilar and coronary arteries, 10–100 μM EPA inhibited the contractions induced by U46619 and shifted the CRCs for U46619 to the right (Fig. 7a,c). As for the basilar arteries, the slope of the regression line for the Schild plot of 10–30 μM EPA versus U46619 was 1.17 (95% confidence interval: 0.16–2.18), which was not significantly different from unity (Fig. 7b). The p*A*_2_ value of EPA versus U46619 for the basilar arteries was 4.90 (95% confidence interval: 4.68–6.03). However, the slope of the regression line for the Schild plot of 30–100 μM EPA versus U46619 was 2.20 (95% confidence interval: 1.17–3.24), which was significantly larger than unity (Fig. 7b). As for the coronary arteries, the slope of the regression line for the Schild plot of 10–100 μM EPA versus U46619 was 1.27 (95% confidence interval: 0.70–1.83), which was not significantly different from unity (Fig. 7d). The p*A*_2_ value of EPA versus U46619 for the coronary arteries was 4.49 (95% confidence interval: 4.28–4.69).

#### Effects of eicosanoic acid (EA) and docosapentaenoic acid (DPA) on U46619-induced responses

**Figure 8:**
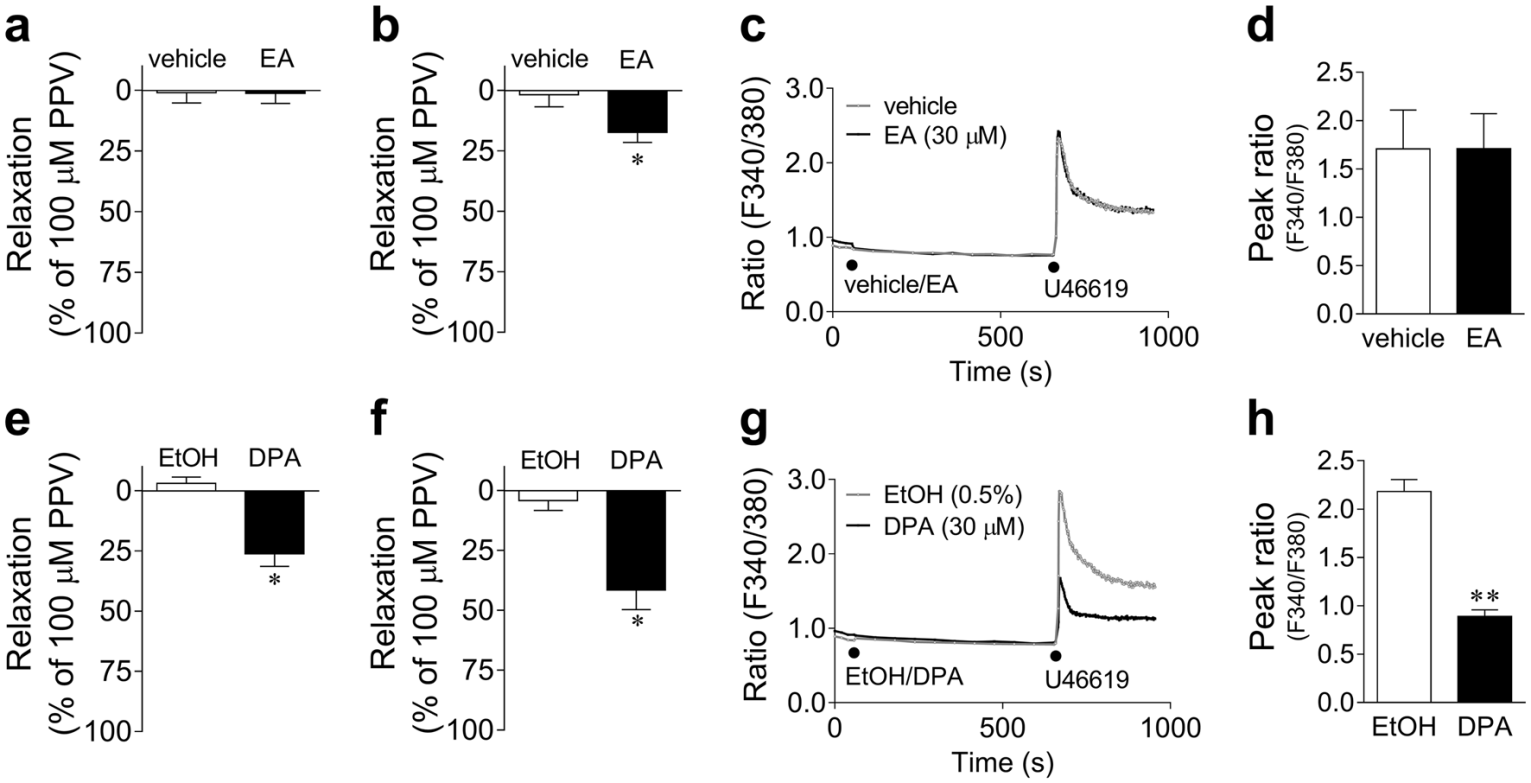
Quantified data of relaxant responses to 30 μM eicosanoic acid (EA) (**a**,**b**) and 30 μM docosapentaenoic acid (DPA) (**e**,**f**) on the contractions induced by 10–30 nM U46619 for porcine basilar (**a,e**) and coronary (**b**,**f**) arteries. Changes in Fura-2 fluorescence intensity ratio (F340/380) induced by 10 nM U46619 (**c,g)** in the presence of 30 μM EA or its vehicle (0.05% dimethylformamide/0.45% EtOH) (**c**) or 30 μM DPA or its vehicle (0.5% EtOH) (**g**) in TP-293T cells; quantified data of the F340/380 peak ratio after application of U46619 (**d**,**h**). Changes in F340/380 are presented as means only (**c,g**), and quantified data are presented as mean ± SEM [n = 6 (**a**), n = 5 (**b**), n = 10 (**c**,**d**), n = 7 (**e**), n = 6 (**f**), and n = 10 (**g**,**h**)]. ●: each drug application. *P < 0.05, **P < 0.01 versus EtOH or vehicle (Student's *t*-test). PPV: papaverine; EtOH: ethanol.

Figure 8 shows the effects of EA (30 μM) (Fig. 8a–d) and DPA (30 μM) (Fig. 8e–h) on U46619-induced responses in porcine arteries and TP-293T cells. EA had no effect on U46619-induced contractions in the basilar artery (Fig. 8a) and Ca^2+^ increases in the TP-293T cells (Fig. 8c,d). In contrast, in the coronary artery, EA slightly but significantly suppressed U46619-induced contractions (Fig. 8b) at ~ 20% of the EPA-induced inhibition. DPA showed stronger inhibitory effects than EA against U46619-induced contractions in both basilar and coronary arteries (Fig. 8e,f). However, the degree of inhibition with DPA (26.1%/41.6% for basilar artery/coronary artery) was 30–50% compared to that with EPA (DPA/EPA = 31.5% for basilar artery; DPA/EPA = 51.0% for coronary artery). DPA largely suppressed the U46619-induced increases in intracellular Ca^2+^ concentration in TP-293T cells (Fig. 8g,h). However, the degree of inhibition with DPA was less than that with EPA (DPA: 59.2%; EPA: 77.4%).

## Discussion

We conducted this study to elucidate the inhibitory effects of EPA on contractions induced by U46619 and PGF_2α_ in porcine cerebral and coronary arteries from isolated tissues where arterial spasms are frequently generated. In addition, possible inhibitory effects of EPA on intracellular Ca^2+^ concentration increases induced by U46619 and PGF_2α_ were investigated in cells expressing human TP receptors; this experiment was performed to determine whether the TP receptor is targeted by the inhibitory effects of EPA against contractions induced by U46619 and PGF_2α_. We found that EPA strongly suppressed the contractions induced by U46619 and PGF_2α_ in the porcine cerebral and coronary arteries. We also successfully showed that the inhibitory effects of EPA on TP receptors are involved in the inhibitory effects of EPA against U46619 and PGF_2α_.

The inhibitory effects of EPA against porcine basilar and coronary artery contractions induced by U46619 and PGF_2α_ likely involve the antagonistic action of this n-3 PUFA on the TP receptor. The rationale for this conclusion is as follows: (1) EPA strongly suppressed intracellular Ca^2+^ concentration increases induced by U46619 and PGF_2α_ in TP-293T cells without affecting PGF_2α_-induced Ca^2+^ concentration increase in FP-293T cells. These results indicate that EPA selectively suppresses either the TP receptor itself or the TP receptor-stimulated intracellular events. (2) EPA competitively suppressed the contractions induced by U46619 in both basilar and coronary arteries, although this inhibition was limited to low concentration ranges in the basilar arteries. (3) U46619 is a stable analog of TXA_2_^14^. The concentrations (10–30 nM for tonic contractions and F340/380 measurements) of U46619 employed in this study were sufficiently lower than the K_i_ values corresponding to all other prostanoid receptors^15^. Furthermore, the contractions induced by U46619 were almost completely inhibited by 1 μM SQ 29,548 in both basilar and coronary arteries: basilar arteries, 93.4 ± 2.2% (mean ± SEM); coronary arteries, 100.2 ± 1.1% (mean ± SEM). The concentration of SQ 29,548 (1 μM), a receptor antagonist with a high selectivity against the TP receptor, used in this study would preclude the interaction of SQ 29,548 with non-TP receptors^15^. Therefore, the contractions induced by U46619 (10–30 nM) in both the basilar and coronary arteries were judged to be almost completely mediated through TP receptors. The finding that EPA showed a marginal effect or no effect on the remaining contractile component in the presence of 1 μM SQ 29,548 suggests that the effects of EPA and those of SQ 29,548 are largely shared. (4) The contractions induced by PGF_2α_ were inhibited by 1 μM SQ 29,548 by ~ 40–70%: basilar arteries, 41.2 ± 6.4% (mean ± SEM); coronary arteries, 73.3 ± 2.1% (mean ± SEM). Therefore, the TP receptor contribution was ~ 40–70% for the PGF_2α_-induced contractions, although the contribution seemed to be larger for coronary arteries than for basilar arteries. In contrast, EPA suppressed PGF_2α_-induced contractions by ~ 70–80%: basilar arteries, 77.2 ± 8.5%; coronary arteries, 68.9 ± 3.6%. Thus, the contribution of the TP receptor seemed to be substantial for the EPA-induced inhibitory effects against the PGF_2α_-induced contractions in both arteries. However, EPA inhibited more strongly than SQ 29,548 in the basilar arteries, while EPA inhibition was almost equal to SQ 29,548 inhibition in the coronary arteries. (5) EPA did not show strong inhibitory effects against the contractions induced by other stimulators (KCl, ACh, His, and 5-HT) in the coronary arteries. (6) EPA was reported to inhibit the [^3^H]SQ 29,548 and [^3^H]U46619 specific binding to human platelets in a concentration-dependent manner^16,17^. Regarding the inhibitory effects of EPA on TP receptor-mediated responses, EPA itself is speculated to play a role because all tested inhibitors of plausible EPA-related metabolite enzymes did not affect the EPA-induced inhibitions; the inhibition by EPA of U46619-induced Ca^2+^ increases in TP-293T cells were still substantial even in the presence of a lipoxygenase inhibitor (nordihydroguaiaretic acid: NDGA), a cytochrome P450 inhibitor (SKF 525A), and a cyclooxygenase inhibitor (indomethacin) (Supplementary Fig. S2).

The relative inhibitory potencies of EPA and DHA against U46619-induced contractions will be discussed next. Previously, we found that DHA, another n-3 PUFA, strongly suppressed contractions induced by U46619 and PGF_2α_ in porcine basilar and coronary arteries, and that TP receptor antagonism is substantially involved in those DHA inhibitory activities^13^. Furthermore, we previously calculated the p*A*_2_ value of DHA versus U46619 from the Schild plot analysis that was performed for the CRCs of U46619 in the absence and presence of DHA; the p*A*_2_ value of DHA was 5.16 in the coronary arteries and 5.44 in the basilar arteries, although the latter value was a reference value since the slope of the Schild plot was > 1.0^13^. In contrast, the present study indicates that the p*A*_2_ value of EPA was 4.49 in the coronary arteries and 4.90 in the basilar arteries. Based on these p*A*_2_ values, the TP receptor antagonistic actions of EPA are estimated to be ~ 3–5-fold (4.7 for coronary arteries, 3.5 for basilar arteries) less potent than those of DHA. A less potent inhibitory action of EPA against DHA was also demonstrated by the results of platelet aggregation assays using U46619^16^ and receptor binding experiments using [^3^H]U46619^17^; the comparison of IC_50_ values showed that EPA was 2–4-fold less potent than DHA (IC_50 (EPA)_/IC_50 (DHA)_ = 2.4–3.7), which is consistent with our current results. EA, which has the same number of carbon atoms as EPA (C = 20) but is a saturated fatty acid, showed no inhibitory effects or only a slight inhibitory effect against U46619-induced responses in basilar/coronary arteries and TP receptor-expressing cells. These results suggest that the presence of carbon–carbon double bonds plays an extremely important role in the EPA-induced inhibitory effects against TP receptor-mediated responses. To examine the possible significance of the number of carbon–carbon double bonds, the inhibitory effects of DPA were also studied. DPA has the same number of carbon atoms as DHA (C = 22) and the same number of carbon–carbon double bonds as EPA (five carbon–carbon double bonds). Thus, DPA was expected to show almost the same inhibitory effect as EPA. However, the inhibitory effects of DPA were smaller than those of EPA; the inhibitory ratio of DPA/EPA was 30–50% in basilar/coronary arteries and ~ 75% in TP-293T cells. Therefore, factors other than the number of carbon atoms and carbon–carbon double bonds could be involved. Comparing the chemical structures of DHA, EPA, and DPA (Supplementary Fig. S3), the position of the first double bond from the carboxyl group is farther for DPA than for DHA and EPA (DPA: 7; DHA: 4; EPA: 5). The difference in inhibitory potencies between DHA/EPA and DPA may reflect the difference in the starting position of the carbon–carbon double bond from the carboxyl group. The difference in inhibitory potencies with DHA and EPA might be due to the difference in the number of double bonds. However, this issue should be further investigated in the future.

Actions other than TP receptor antagonism could underlie the mechanisms by which EPA inhibits prostanoid-induced arterial contractions. In particular, these mechanisms seem to be more substantial in cerebral arteries because of the following results. (1) In both basilar and coronary arteries, the contractile components of the PGF_2α_-induced contraction remaining after treatment with 1 μM SQ 29,548 were further inhibited by 30 μM EPA, and the additional inhibitory effects of EPA were more remarkable in the basilar arteries (coronary arteries, 16.0%; basilar arteries, 35.5%). (2) The slope of the regression line of the Schild plot was significantly larger than unity with high EPA concentrations (≥ 30 μM) in the cerebral arteries, while the slope was unity in the coronary arteries. This suggests that the targets of EPA include non-TP receptor sites in addition to the TP receptor. A candidate for such non-TP receptor targets of EPA would include K^+^ channels functionally coupled with the TP receptor; such K^+^ channels might be activated by EPA itself or its active metabolites. This speculation can be deduced from the finding that in both coronary and basilar arteries, EPA did not suppress the contraction induced by a high KCl level (80 mM), which cannot be counteracted by possible K^+^ channel opening. The finding that EPA did not suppress the contraction due to high KCl levels also suggests that voltage-dependent L-type Ca^2+^ channels are unlikely targets of EPA, at least in porcine basilar and coronary arteries.

In clinical studies, EPA monotherapy was reported to be effective against cardiovascular diseases, while combination therapy of EPA/DHA did not show significant effects^2^. Therefore, the plausible superior effectiveness of EPA to combination treatments of EPA/DHA was examined in our contraction studies. However, in both basilar and coronary arteries, the inhibitory effects against U46619-induced contractions were not significantly different between EPA treatment alone and EPA/DHA combination treatment (Supplementary Fig. S4). Therefore, the differences reported in clinical studies cannot be supported by the immediate effects of these fatty acids on the isolated arteries. To explain the difference between experimental and clinical studies, factors other than inhibitory effects against TP receptor-mediated responses should be considered.

The important roles of TXA_2_ in vasospasm and our results suggest that an active intake of EPA is expected to prevent the onset of cerebral and coronary arterial diseases associated with increased TXA_2_, especially in patients at high-risk of cardiovascular diseases, including dyslipidemia and hypertension.

## Methods

### Preparation of basilar and coronary artery strips from pigs

Porcine brains and hearts were obtained from Tokyo Shibaura Zoki Co., Ltd. (Tokyo, Japan) and transported to our laboratory in ice-cold Krebs-HEPES (2-[4-(2-hydroxyethyl)-1-piperazinyl]ethanesulfonic acid) solution (126.9 mM NaCl, 5.9 mM KCl, 2.36 mM CaCl_2_, 1.18 mM MgCl_2_, 20 mM HEPES, and 11.8 mM glucose). Upon arrival, the basilar and coronary arteries were isolated carefully, as previously described^13^. The basilar arteries were sliced into endothelium-intact ring segments (~ 2–3 mm in length). The coronary arteries were sliced into ring segments (~ 2 mm in length). The coronary ring segments were further opened along the longitudinal axis and the endothelium was removed gently using a cotton swab.

### Recording isometric tension changes in basilar and coronary artery contractions

Recordings of isometric tension changes in basilar and coronary artery contractions were performed as previously described^13^. Briefly, the endothelium-intact basilar artery rings were suspended under a 0.75-g resting tension with stainless steel hooks (150-μm outer diameter) in a 5–6-mL organ bath containing normal Tyrode's solution (158.3 mM NaCl, 4.0 mM KCl, 2.0 mM CaCl_2_, 1.05 mM MgCl_2_, 0.42 mM NaH_2_PO_4_, 10.0 mM NaHCO_3_, and 5.6 mM glucose). The endothelium-removed coronary artery strips were vertically suspended under a 2.0-g resting tension in a 20-mL organ bath containing normal Tyrode's solution. The Tyrode's solution was continuously bubbled with 95% O_2_:5% CO_2_ and maintained at 35.0 ± 1.0 °C (pH = 7.4). A force–displacement transducer (T7-8-240, Orientec Co., Ltd., Tokyo, Japan; TB-612 T, Nihon Kohden, Tokyo, Japan) and a carrier amplifier (signal conditioner MSC-2, Labo Support Co., Osaka, Japan; AD-632 J/AP-621G/AP-620G, Nihon Kohden) were used to measure isometric tension changes, which were recorded using LabChart™ (Version 7) software and PowerLab™ (ADInstruments, Bella Vista, NSW, Australia). Before recording contractions induced by 80 mM KCl Tyrode's solution (82.3 mM NaCl, 80.0 mM KCl, 2.0 mM CaCl_2_, 1.05 mM MgCl_2_, 0.42 mM NaH_2_PO_4_, 10.0 mM NaHCO_3_, and 5.6 mM glucose), the basilar/coronary preparations were incubated in normal Tyrode's solution for 60/120 min, replenishing the solution every 20 min. Then, the basilar/coronary strips were contracted twice using 80 mM KCl to ensure normal contractile responses from the strips. Subsequent basilar artery experiments were performed in the presence of 100 μM *N*^G^-nitro-l-arginine methyl ester (l-NAME, an inhibitor of nitric oxide synthase), and the strips were further contracted using 80 mM KCl in the presence of 100 μM l-NAME.

To prevent potential effects of endogenous prostaglandins, all tension recordings were performed in the presence of 3 μM indomethacin.

### Effects of EPA on porcine basilar and coronary artery tonic contractions induced by U46619, PGF_2α_, and KCl

After the 80 mM-KCl-induced contractions were recorded thrice/twice and the basilar/coronary artery preparations were equilibrated in normal Tyrode's solution for 30/60 min, these strips were contracted with 1 nM U46619, 3 μM PGF_2α_, or 80 mM KCl. The contractions induced by these concentrations of U46619 and PGF_2α_ were ~ 40–80% of the 80 mM KCl-induced contractions. As for the basilar artery experiments, if the contractions induced by PGF_2α_ and U46619 were clearly lower than the third 80 mM KCl-induced contractions, increased concentrations of PGF_2α_ (≤ 6 μM) and U46619 (≤ 30 nM) were applied to induce contractions that were 40–70% of the third 80 mM KCl-induced contractions. Similarly, for the coronary arteries, if the contractions induced by PGF_2α_ and U46619 were < 40% of the second 80 mM KCl-induced contractions, increased concentrations of PGF_2α_ (≤ 30 μM) and U46619 (≤ 30 nM) were applied to induce contractions ≥ 40% of the third 80 mM KCl-induced contractions.

These arterial contractions were allowed to stabilize. Then, 1–30 μM EPA, 30 μM EA/DPA, an EPA and DHA combination (each 5 μM for basilar arteries or 3 μM for coronary arteries), or their vehicle (0.1% ethanol (EtOH) for EPA, DPA, and EPA/DHA combination; 0.05% dimethylformamide/0.45% EtOH for EA), was applied to the bath solution. The EPA concentrations were applied cumulatively for the basilar artery experiments and individually for the coronary artery experiments. When these arterial relaxations stabilized or occurred for ≥ 40 min, papaverine (PPV, 100 μM) was applied to further relax the preparations. The EPA-induced relaxation was calculated relative to the tension level before application (0% relaxation) and the stable tension level after PPV application (100% relaxation).

As for the SQ 29,548 experiments, after the arterial contractions stabilized, 1 μM SQ 29,548 was applied and the SQ 29,548-induced relaxation was allowed to reach stability. Then, 30 μM EPA was applied, and the strips were relaxed with 100 μM PPV. The SQ 29,548- and SQ 29,548 plus EPA-induced relaxations were calculated relative to the stable tension level before application (0% relaxation) and the tension level before PGF_2α_/U46619 application (100% relaxation).

In the CRC experiments, after recording contractions induced by 80 mM KCl, 0.1% EtOH or 10–100 μM EPA was applied 40 min before U46619 application, and U46619 was administered cumulatively.

### Effects of EPA on ACh-, His-, and 5-HT-induced porcine coronary artery phasic contractions

After recording contractions induced by 80 mM KCl, the coronary strips were contracted for 10 min with 3 μM ACh, 10 μM His, or 30 μM 5-HT three times at 60 min intervals. EtOH (0.1%) or 3–30 μM EPA was applied 30 min before the third contraction induced by each drug. The maximum value of the third contraction induced by each drug in the presence of EtOH or EPA was calculated relative to the maximum value of the second contraction (control, 100%).

### Measurement of increases in F340/380 induced by U46619 and PGF_2α_ in TP-293T/FP-293T cells

Increases in F340/380 in TP-293T/FP-293T cells were performed as previously described^13^. Briefly, the cells^13^ were cultured in Dulbecco's modified Eagle's medium (DMEM) supplemented with 10% heat-inactivated fetal bovine serum in a humidified incubator at 37 °C and 5% CO_2_. The day before F340/380 measurements, the cells were seeded at ~ 90% confluence in a 96-well plate. The next day, the DMEM was exchanged with 5 μM Fura-2 AM-containing medium, and Fura-2 AM was loaded into the cells for 30–60 min at 37 °C and 5% CO_2_. Afterward, the cells were rinsed with Fura-2 AM-free medium, and their F340/380 values were measured with a microplate reader (Infinite F200 Pro, Tecan Group Ltd., Mӓnnedorf, Switzerland or Nivo, PerkinElmer, Inc., MA, USA) at 25 °C. After the cells were equilibrated for 10 min in the presence of 30 μM EPA/EA/DPA, 1 μM SQ 29,548; 100 μM phloretin; or their vehicle (0.5% EtOH for EPA/DPA/SQ 29,548/phloretin; 0.05% dimethylformamide/0.45% EtOH for EA), 10 nM U46619 or 3 μM PGF_2α_ was applied by the injector module, and F340/380 was measured for 5 min. When using 30 μM NDGA, 30 μM SKF 525A, and 3 μM indomethacin, these inhibitors were added in medium 10 min before EPA/EtOH administration. Increases in the F340/380 ratios were considered relative increases in intracellular Ca^2+^ concentrations. At the end of the experiment, 5 μM ionomycin and 50 mM Mn^2+^ were applied to determine background fluorescence. This background fluorescence was subtracted from the fluorescence intensities of all measurements.

### Drugs

EPA; U46619; SQ 29,548; DPA; NDGA; and ionomycin calcium were obtained from Cayman Chemical Co. (Ann Arbor, MI, USA). Dinoprost (PGF_2α_) was purchased from Fuji Pharma Co. Ltd. (Tokyo, Japan). Histamine dihydrochloride and indomethacin were obtained from Sigma-Aldrich Co. LLC (St. Louis, MO, USA). ACh chloride was acquired from Daiichi Sankyo Co., Ltd. (Tokyo, Japan). 5-HT-creatinine sulfate monohydrate and PPV hydrochloride were obtained from FUJIFILM Wako Pure Chemical Co. (Tokyo, Japan). l-NAME hydrochloride and Fura-2 AM and were acquired from DOJINDO Laboratories (Kumamoto, Japan). SKF 525A hydrochloride was obtained from Biomol GmbH (Hamburg, Germany). EA and phloretin were obtained from Tokyo Chemical Industry Co., Ltd. (Tokyo, Japan). The other chemicals were of reagent grade and were obtained from generic suppliers.

U46619 was dissolved in 100% EtOH as a 10 mM stock solution for cumulative applications and 70% EtOH as a 100 μM stock solution for single applications. SQ 29,548/phloretin were dissolved in 100% EtOH as 2 mM/6 mM stock solutions. EPA/DPA were dissolved in 100% EtOH as 6 mM/3 mM stock solutions for the F340/380 measurements. EPA/DHA and DPA were dissolved in 10% EtOH as 10 mM and 3 mM stock solutions, which were diluted with 10% EtOH for the vascular experiments. EA was dissolved in dimethylformamide as a 60 mM stock solution, which was diluted with 100% EtOH. Fura-2 AM was dissolved in dimethyl sulfoxide (DMSO) as a 1 mM stock solution. Aqueous stock solutions were prepared for all other drugs, which were diluted with distilled water for experimental use.

### Statistical analysis

The construction of CRCs of contractions generated by U46619 and the Schild plot of EPA versus U46619 were performed using GraphPad Prism™ software (version 6.0; GraphPad Software, Inc., San Diego, CA, USA) as previously described^13^. The peak and AUC values of F340/380 within 5 min after stimulation were calculated with GraphPad Prism. All values are presented as mean ± SEM or mean with 95% confidence intervals of data obtained from different numbers (n) of preparations/experiments. The statistical analyses were performed with GraphPad Prism, and differences among values were verified with Dunnett's test after one-way ANOVA or Student's *t-*tests. Statistical significance was set at P < 0.05.

## Supplementary Information

Supplementary Figures.

## Data Availability

The data that support the findings of this study are available from the corresponding author, K.O., upon reasonable request.
