# Supplementary material for: Eicosapentaenoic acid (EPA)-induced inhibitory effects on porcine coronary and cerebral arteries involve inhibition of prostanoid TP receptors
Source: Sci Rep. 2022 Jul 27;12:12829. doi: 10.1038/s41598-022-16917-6 (PMC9329469; doi:10.1038/s41598-022-16917-6)
Supplement: Supplementary file 1 — Supplementary Figures. [file 41598_2022_16917_MOESM1_ESM.pdf]

Supplementary Figures

Scientific reports / Article

Eicosapentaenoic acid (EPA)-induced inhibitory effects on porcine coronary and cerebral arteries involve inhibition of prostanoid TP receptors

Kento Yoshioka[#], Keisuke Obara[#, *], Shunya Oikawa, Kohei Uemura, Akina Yamaguchi, Kazuki Fujisawa, Hitomi Hanazawa, Miki Fujiwara, Taison Endoh, Taichi Suzuki, Montserrat De Dios Regadera, Daichi Ito, Noboru Saitoh, Yutaka Nakagome, Toma Yamashita, Mayu Kiguchi, Yuka Saito, Yuri Nakao, Hinako Miyaji, Guanghan Ou, Keyue Xu, and Yoshio Tanaka

Department of Chemical Pharmacology, Faculty of Pharmaceutical Sciences, Toho University, Miyama 2-2-1, Funabashi-City, Chiba 274-8510, Japan

[#] These authors contributed equally to this work.

***Correspondence to:**
Keisuke Obara, Ph.D.
Department of Chemical Pharmacology
Faculty of Pharmaceutical Sciences, Toho University
Miyama 2-2-1, Funabashi-City
Chiba 274-8510, Japan.
E-mail: keisuke.obara@phar.toho-u.ac.jp

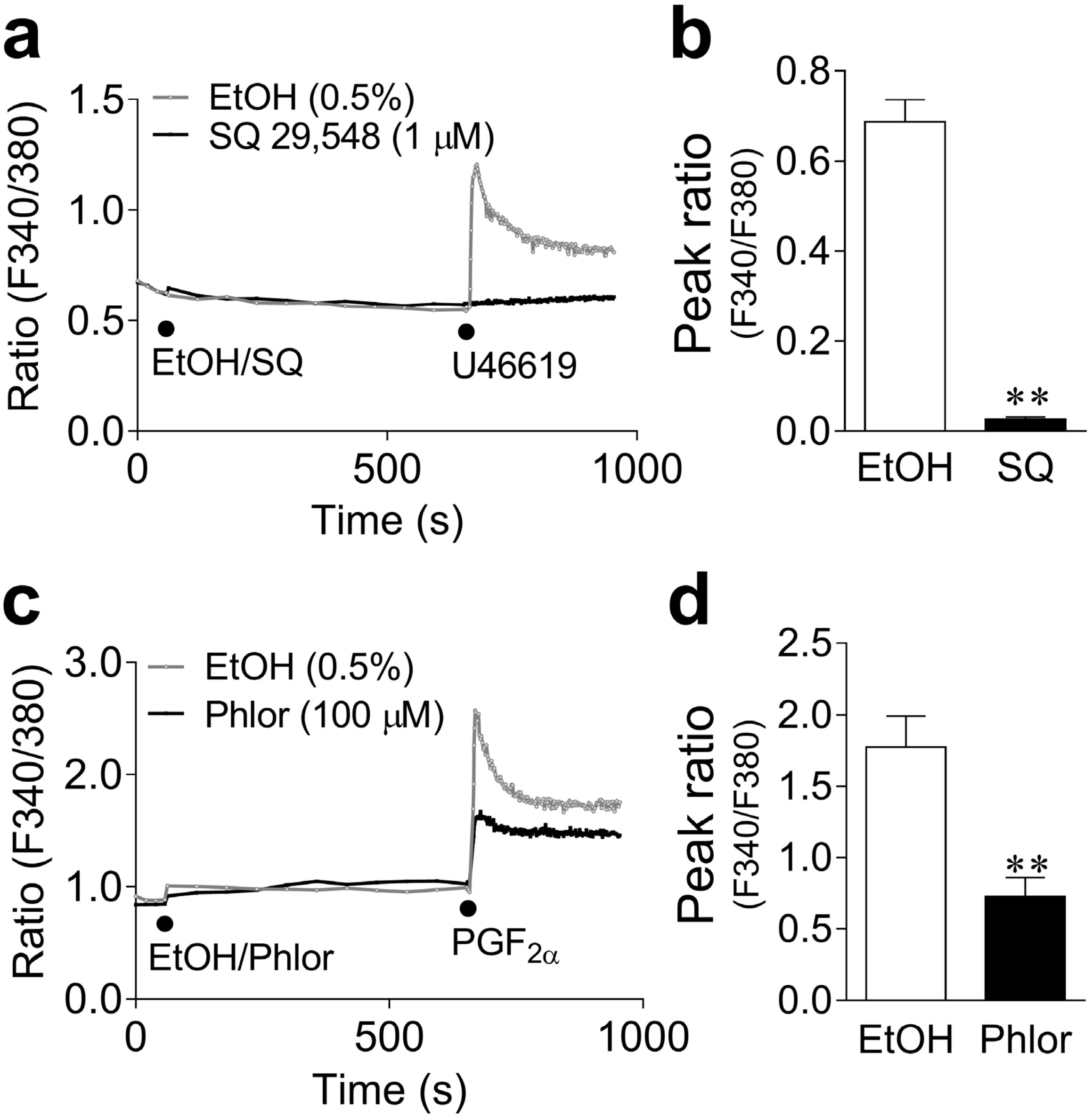

Supplementary Fig. S1

The effects of SQ 29,548 and phroletin on the increases in F340/380 induced by U46619 in TP-293T cells and $PGF_{2\alpha}$ in FP-293T cells. Changes in F340/380 induced by 10 nM U46619 in the presence of 1 μM SQ 29,548 (SQ) or 0.5% ethanol (EtOH) in TP-293T cells (**a**); quantified data of the F340/380 peak after application of U46619 (**b**). Changes in F340/380 induced by 3 μM $PGF_{2\alpha}$ in the presence of 100 μM phloretin (Phlor) or 0.5% EtOH in FP-293T cells (**c**); quantified data of the F340/380 peak after application of $PGF_{2\alpha}$ (**d**). Changes in F340/380 are presented as means only, and quantified data are presented as mean ± SEM [n = 6 (**a**, **b**), n = 18 (**c**, **d**)]. ●: each drug application. **P < 0.01 versus EtOH (Student's *t*-test).

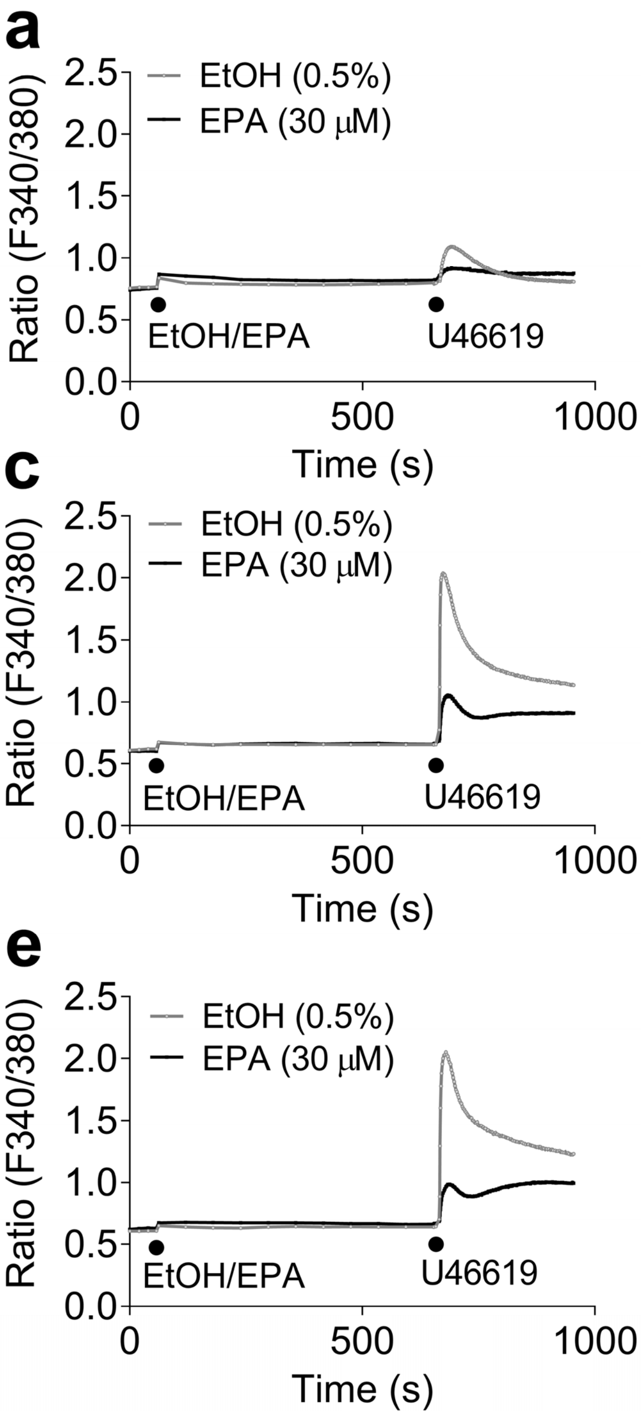

Supplementary Fig. S2

The effects of EPA or its vehicle (0.5% EtOH) on the increases in F340/380 induced by U46619 in TP-293T cells in the presence of various inhibitors of plausible EPA-related metabolite enzymes. Changes in F340/380 induced by 10 nM U46619 in the presence of 30 μM nordihydroguaiaretic acid (**a**, **b**), 30 μM SKF 525A (**c**, **d**), or 3 μM indomethacin (**e**, **f**) in TP-293T cells; quantified data of the F340/380 peak after application of U46619. Changes in F340/380 are presented as means only, and quantified data are presented as mean ± SEM (n = 10 for all). ●: each drug application. **P < 0.01 versus EtOH (Student's *t*-test). EPA: eicosapentaenoic acid; EtOH: ethanol

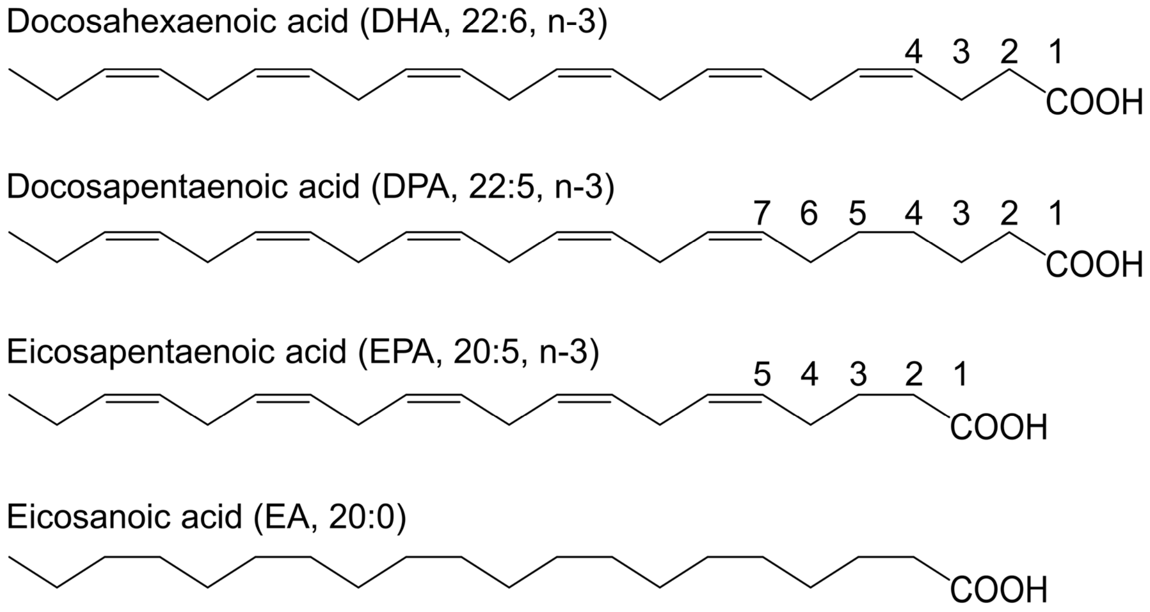

Supplementary Fig. S3

Chemical structures of docosahexaenoic acid, docosapentaenoic acid, eicosapentaenoic acid, and eicosanoic acid. The arabic numerals shown in the chemical structures represent carbon atoms from the carboxylate group.

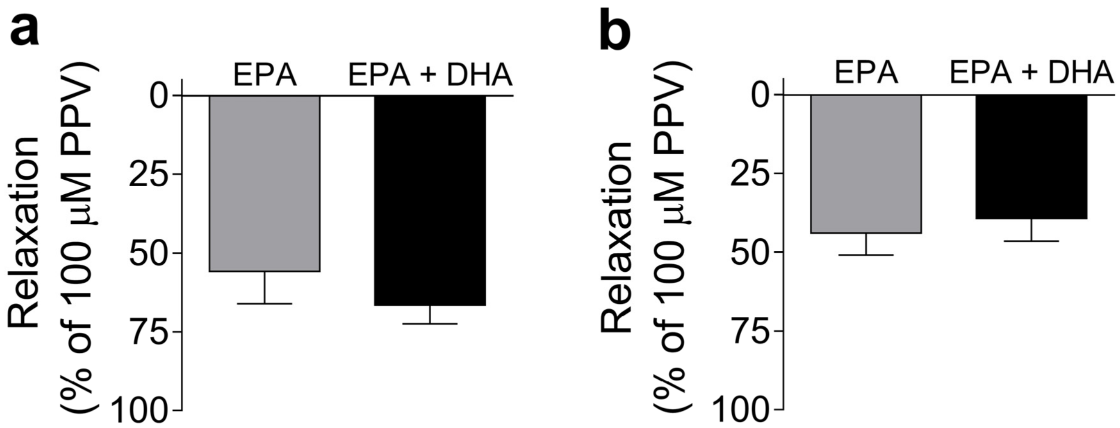

Supplementary Fig. S4

Effects of EPA or combination treatment of EPA/DHA on contractions induced by U46619 in porcine basilar and coronary arteries. **a**: Relaxation by EPA (10 μM) or EPA/DHA (5 μM each) on U46619 (10–30 nM)-induced contractions in basilar arteries. **b**: Relaxation by EPA (6 μM) or EPA/DHA (3 μM each) on U46619 (10–30 nM)-induced contractions in coronary arteries. Data are presented as mean ± SEM [n = 5 (**a**) and n = 8 (**b**)]. PPV: papaverine; EPA: eicosapentaenoic acid; DHA: docosahexaenoic acid
